# Similarities between the Binding Sites of SB-206553 at Serotonin Type 2 and Alpha7 Acetylcholine Nicotinic Receptors: Rationale for Its Polypharmacological Profile

**DOI:** 10.1371/journal.pone.0134444

**Published:** 2015-08-05

**Authors:** Patricia Möller-Acuña, J. Sebastián Contreras-Riquelme, Cecilia Rojas-Fuentes, Gabriel Nuñez-Vivanco, Jans Alzate-Morales, Patricio Iturriaga-Vásquez, Hugo R. Arias, Miguel Reyes-Parada

**Affiliations:** 1 Centro de Bioinformática y Simulación Molecular, Facultad de Ingeniería, Universidad de Talca, 2 Norte 685, Casilla 721, Talca, Chile; 2 Programa de Doctorado en Biotecnología, Universidad de Santiago de Chile, Santiago, Chile; 3 Laboratorio de Biología Computacional, Fundación Ciencia & Vida, Santiago, Chile; 4 Facultad de Ingeniería y Ciencias, Universidad de la Frontera, Temuco, Chile; 5 Department of Medical Education, California Northstate University College of Medicine, Elk Grove, CA, United States of America; 6 Escuela de Medicina, Facultad de Ciencias Médicas, Universidad de Santiago de Chile, Santiago, Chile; 7 Facultad de Ciencias de la Salud, Universidad Autónoma de Chile, Talca, Chile; Russian Academy of Sciences, Institute for Biological Instrumentation, RUSSIAN FEDERATION

## Abstract

Evidence from systems biology indicates that promiscuous drugs, i.e. those that act simultaneously at various protein targets, are clinically better in terms of efficacy, than those that act in a more selective fashion. This has generated a new trend in drug development called polypharmacology. However, the rational design of promiscuous compounds is a difficult task, particularly when the drugs are aimed to act at receptors with diverse structure, function and endogenous ligand. In the present work, using docking and molecular dynamics methodologies, we established the most probable binding sites of SB-206553, a drug originally described as a competitive antagonist of serotonin type 2B/2C metabotropic receptors (5-HT_2B/2C_Rs) and more recently as a positive allosteric modulator of the ionotropic α7 nicotinic acetylcholine receptor (nAChR). To this end, we employed the crystal structures of the 5-HT_2B_R and acetylcholine binding protein as templates to build homology models of the 5-HT_2C_R and α7 nAChR, respectively. Then, using a statistical algorithm, the similarity between these binding sites was determined. Our analysis showed that the most plausible binding sites for SB-206553 at 5-HT_2_Rs and α7 nAChR are remarkably similar, both in size and chemical nature of the amino acid residues lining these pockets, thus providing a rationale to explain its affinity towards both receptor types. Finally, using a computational tool for multiple binding site alignment, we determined a consensus binding site, which should be useful for the rational design of novel compounds acting simultaneously at these two types of highly different protein targets.

## Introduction

Observations coming from systems biology increasingly indicate that “promiscuous” drugs, *i*.*e*. targeting multiple receptors, might show better profiles than selective compounds, regarding both efficacy and side effects [1–4]. This has generated a new trend in drug design and development called polypharmacology [4]. However, the rational design of these polypharmacological agents is a difficult task, particularly if the addressed targets exhibit high structural or functional diversity.

Drug-receptor interaction relies primarily on the shape and electronic complementarities between the ligand and the receptor’s binding site. Therefore, it seems reasonable to assume that all proteins targeted by a given compound should have certain similarities of these features at their binding sites. In fact, approaches based on this idea are being used to predict polypharmacology for agents acting at highly different proteins [5,6].

**Fig 1:**
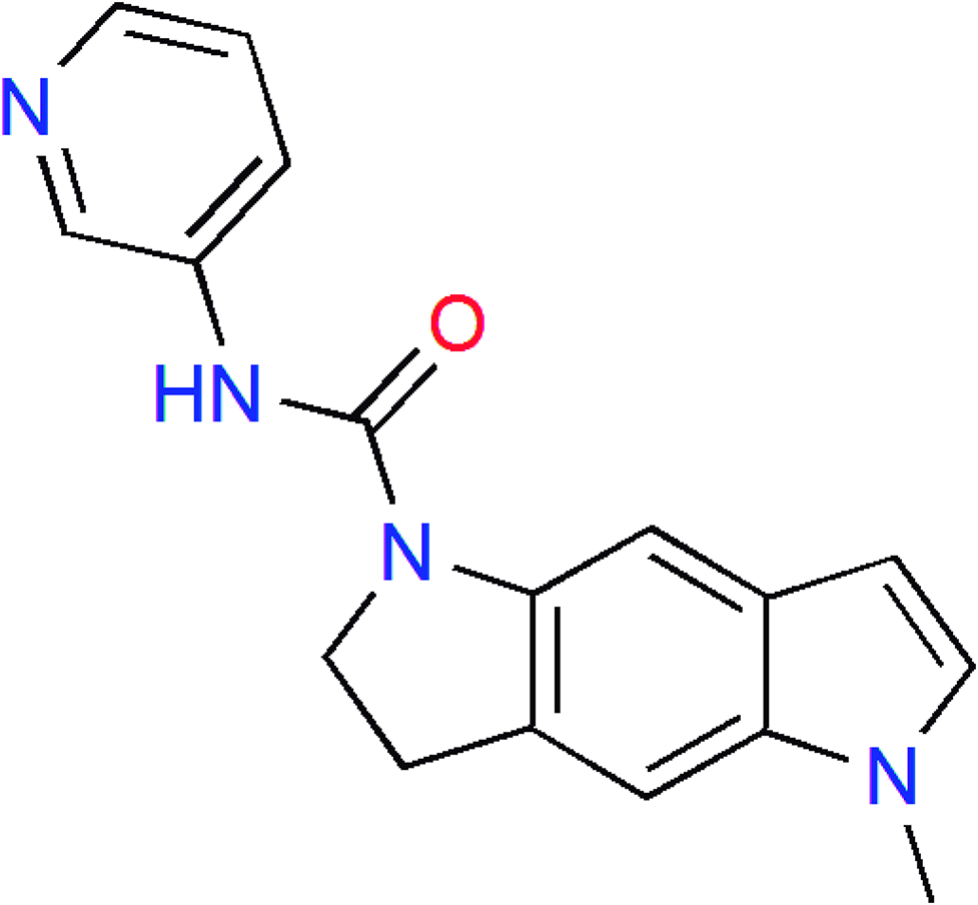
Chemical structure of SB-206553.

SB-206553 (Fig 1) is a dihydropyrroloindole derivative described two decades ago as a competitive antagonist of metabotropic serotonin type 2B/2C receptors (5-HT_2B/2C_Rs), exhibiting anxiolytic properties [7,8]. Later it was reclassified as a 5-HT_2_R inverse agonist [9]. More recently, this compound was shown to act also as a positive allosteric modulator at the ionotropic α7 nicotinic acetylcholine receptor (nAChR) [10].

5-HT_2B/2C_Rs are closely related G-protein-coupled receptors (GPCR), whose stimulation activates phospholipase-C and phospholipase-A_2_-mediated responses [11]. The crystal structure of the 5-HT_2B_R bound to ergotamine was recently described [12], allowing a detailed analysis of the structural requirements necessary for ligand binding.

On the other hand, the α7 nAChR is a member of the pentameric ligand-gated ion channel superfamily (pLGIC), whose activation allows Ca^2+^ influx at levels ~ 10–20 times higher than for Na^+^ influx, and concomitantly K^+^ efflux [13]. Although the crystal structure for this homomeric ionotropic receptor has not been reported yet, it can be reliably modeled using the acetylcholine binding protein (AChBP) and/or the muscular nAChR structures as templates [14].

Considering that the comparative analysis of the binding sites/modes of drugs showing affinity at diverse receptors could facilitate the future design of novel multitarget ligands, in the present work we first determined the most important components at each putative binding site and the possible binding modes of SB-206553 at the 5-HT_2B_, 5-HT_2C_, and α7 nACh receptors, respectively. Then, using a statistical algorithm, the similarity between these binding sites was determined. Our analysis show that the most plausible binding sites for SB-206553 at the 5-HT_2_ and α7 nACh receptors are remarkably similar, both in size and in the chemical nature of the amino acid residues lining these pockets, thus providing a rationale to explain the affinity showed by the drug upon these two highly different protein targets.

## Materials and Methods

### Homology modeling of α7 and 5-HT_2C_ receptors

The models were prepared using MODELLER v9.9 [15]. The extracellular domain (ECD) of the α7 nAChR was built using the crystallographic structure of the *Lymnaea stagnalis* AChBP complexed with nicotine (PDB code 1UW6; [16]) as template, whereas the transmembrane domain (TMD) was built on the basis of the structure of the *Torpedo marmorata* nAChR (PDB code 2BG9; [17]). The amino acid sequence of the α7 nAChR was aligned with those from the AChBP or *Torpedo* nAChR using ClustalW [18], obtaining 24% and 45% of identity (38% and 57% of similarity), respectively.

Initially, 50 models of each subunit (both in the ECD and the TMD) were generated, and those showing the lowest values of molpdf (Modeller objective function) and DOPE (Discrete Optimized Protein Energy) were chosen for further validation. Subunit assembly (ECD and TMD separately), was performed with the ICM software [19] using the AChBP or *Torpedo* nAChR as templates. All other modeling conditions were as previously described [20, 21] (see below).

For the modeling of the 5-HT_2C_R, we used the crystal structure of the 5-HT_2B_R linked to the chimeric protein BRIL (PDB code 4IB4; [12]) as template. The amino acid sequences of both serotonergic receptors were aligned with ClustalW, yielding 76% identity. All other modeling conditions were as previously described [22,23]. Briefly, the best α7 and 5-HT_2C_ receptor models were stereochemically and energetically evaluated by the ANOLEA web service [24] and with PROCHECK [25]. Missing hydrogen atoms, bond orders and disulfide bonds were added to the receptor models, and also to the 5-HT_2B_R structure, using the "Protein Preparation Wizard" module [26] included in the Schrödinger Maestro suite. Then, protein structures (as appropriate) were embedded in a hydrated palmitoyl-oleyl-phosphatidyl-choline (POPC) bilayer membrane, solvated in a water box (SCP water model), and ions were added creating an overall neutral system. Ionic strength was fixed to approximately 0.12 M NaCl, according to experimental data [27]. The final systems were subjected to a molecular dynamics (MD) simulation for 5 ns using Desmond software from Schrödinger Maestro [28]. The isobaric-isothermal ensemble (NPT, temperature of 310 K and 1 atm) was used to perform MD calculations. The equations of motion were integrated using a time step of 2 fs. The simulation time was sufficient to obtain an equilibrated system (root mean square deviation-RMSD- values <2 Å).

### Docking

The SB-206553 structure was obtained from PubChem [29] and optimized using MOPAC2012 [30] with the PM6 semiempirical method.

To define the probable binding site(s) for SB-206553 in the α7 nACh, 5-HT_2C_, and 5HT_2B_ receptors, a blind docking centered on the target macromolecule was performed using the AutoDock suite 4.2 [31]. It should be noted that in the case of the α7 nAChR, the molecular docking was performed separately in the ECD and TMD. The docking procedure was applied to the whole protein/domain, without defining or imposing a unique binding site. A grid box, large enough to accommodate free motion of the drug (126 Å^3^ with a grid-point spacing of 0.375 Å), was built (using AutoDockTools) in each case. The grid maps were calculated using AutoGrid 4.0. All other docking conditions were as previously described [22,32], except for the number of generations (200), energy evaluations (25,000,000), and docking runs (150), which were set according to Galeazzi *et al*. [33]. The docked compounds forming the receptor-drug complexes were built using the lowest docked-energy binding positions. Then, these conformations were used for further production MD simulations.

### Molecular dynamics

The protein/domain-ligand complexes with the highest affinity (inferred from docking energy) were submitted to MD simulations to evaluate the stability of each complex and, in the case of the α7 nAChR, to define the most probable binding site for SB-206553.

For this purpose, the receptor-ligand complexes were prepared with Desmond (System builder module) included in the Schrödinger Maestro suite [28]. In the case of SB-206553 docked to the α7 nAChR-ECD, the complex was inserted into an orthorhombic water box with SPC solvent model (85 Å x 95 Å x 60 Å). On the other hand, complexes of SB-206553 with the α7 nAChR-TMD, 5HT_2B_R or 5HT_2c_R were embedded in a POPC membrane and solvated in an orthorhombic water box with the SPC solvent model (80 Å x 190 Å x 69 Å for the α7 nAChR-TMD; and 121 Å x 130 Å x 97 Å for the 5-HT_2_Rs). In all cases, the systems were neutralized with Na^+^ ions and a salt concentration of 0.12 M NaCl was used to mimic physiological conditions.

The final systems were subjected to 10-ns MD simulations, using previously described conditions [34] with minor modifications. Briefly, for each system, an NPT ensemble (temperature = 310 K, pressure = 1 atm, using the Langevin Piston method), a time step of 2 fs and the OPLS2005 force field were used. The Particle-Mesh Ewald (PME) method was used to compute long-range electrostatic interactions, whereas van der Waals interactions were smoothly switched off at 9 Å. This procedure was performed with the Molecular Dynamics module of Desmond included in the Schrödinger Maestro suite [28]. The RMSD values for the position of atoms in the simulated systems were utilized to appraise the stability of the ligand in the binding site throughout the trajectory of the MD. Visualization of protein-ligand complexes and analysis of MD trajectories was done using the VMD software package [35].

### Similarity measurements

The similarity of the SB-206553 binding sites at the different evaluated receptors was determined by using the PocketMatch algorithm [36]. Binding site comparisons were performed using the procedure described by the algorithm’s authors with minor modifications [37]. Briefly, each binding site was represented as a sorted list of distances between the amino acids present at a given distance from the ligand docked in each protein. To do this, each residue was classified into one of five groups, considering its chemical properties. Then, each residue was represented as a set of three points corresponding to the coordinates of the alpha C, the beta C and the centroid of the side chain. The distances between these three points of each residue and each of the three points in every other residue in the binding sites were measured and sorted in ascending order. The sorted and organized distances were aligned and compared using a threshold of 0.5 Å, which was established considering the natural dynamics of biological systems. The similarity between sites, referred to as the PMScore, was measured by scoring the alignment of the pair of sites under comparison. Thus, the PMScore represents the percentage of the number of “matches” calculated over the maximal number of distances computed for each binding site. A PMScore of 0.5 (50%) or higher was considered as indicative of similarity between binding sites. A script allowing the automatic evaluation of PMScores considering distances from 3.5 Å to 8 Å from the ligand was developed. Thus, we built similarity profiles that graphically show at what distances from the ligand (if any) the binding sites are similar.

### Binding sites alignment and common binding site generation

Structural alignments of the binding sites of SB-206553 at the 5-HT_2_ and α7 nACh receptors were performed using the MultiBind computational method [38]. This approach reveals the common physicochemical patterns that may be responsible for the binding of the same ligand to different protein targets. For the recognition of common patterns, MultiBind performed a multiple alignment between the binding sites defined by all residues of the 5-HT_2_ and α7 nACh receptors that were located up to 4 Å from SB-206553. Multiple structural rearrangements of superimposed binding sites were subsequently performed using a Geometric Hashing technique [39]. Briefly, this method consists of two main processes: a) pre-processing of the features of each binding site conformations and hashing them into a table; and b) recognition of the similar features in the objects of the hash table. In the pre-processing, each amino acid was denoted by pseudo centers (X, Y, and Z coordinates), which provide a singular physicochemical property to the binding site: hydrogen-bond donor, hydrogen-bond acceptor, mixed donor/acceptor, hydrophobic aliphatic or aromatic contacts. Finally, MultiBind performed a combination of multiple superimposed binding site conformations, in order to find consensus binding patterns. Then, the highest scored consensus-binding site conformations at the 5-HT_2_ and α7 nACh receptors were manually depurated. Here, to generate a unique and common binding site, the equivalent amino acids (same physicochemical group: polar, non-polar, positively or negatively charged) that appeared superimposed in all three binding sites were merged. On the contrary, the non-equivalent amino acids in all three binding sites were preserved in the final consensus-binding site.

## Results and Discussion

### Homology models

**Fig 2:**
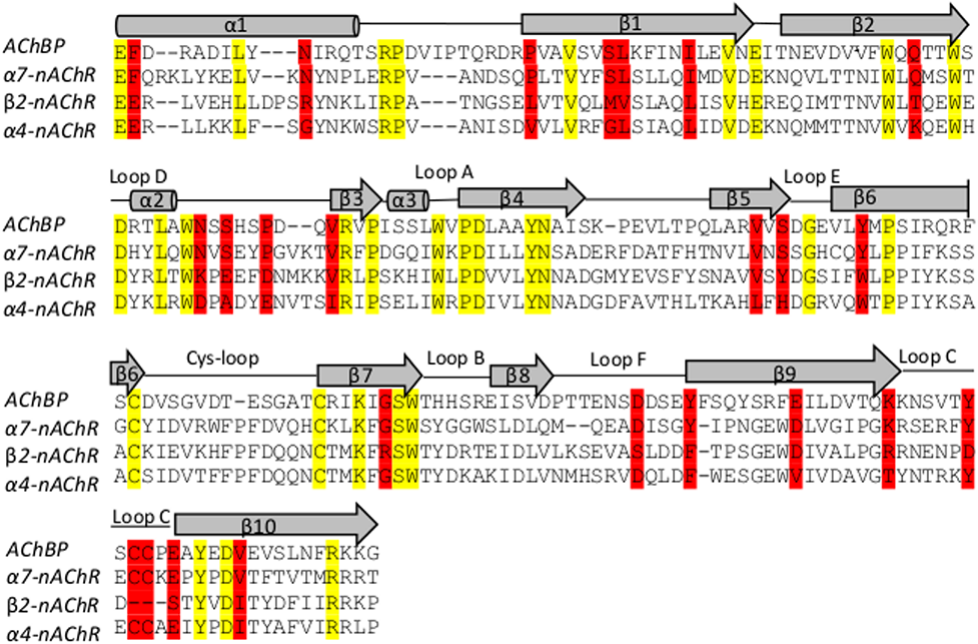
Alignment of the extracellular (ECD) α7, α4 and β2 nAChR subunits and AChBP sequences using ClustalW. Conserved residues are highlighted in yellow and partially conserved residues highlighted in red. Secondary structures are shown schematically above the sequences; alpha helices and beta sheets are represented by cylinders and arrows respectively.

**Fig 3:**
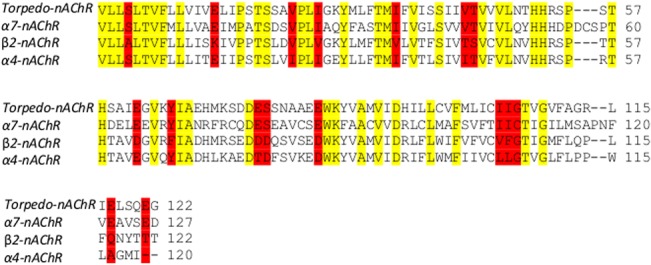
Alignment of the transmembrane (TMD) α7, α4 and β2 nAChR subunits and *Torpedo marmorata* nAChR sequences using ClustalW. Conserved residues are highlighted in yellow and partially conserved residues are highlighted in red.

**Fig 4:**
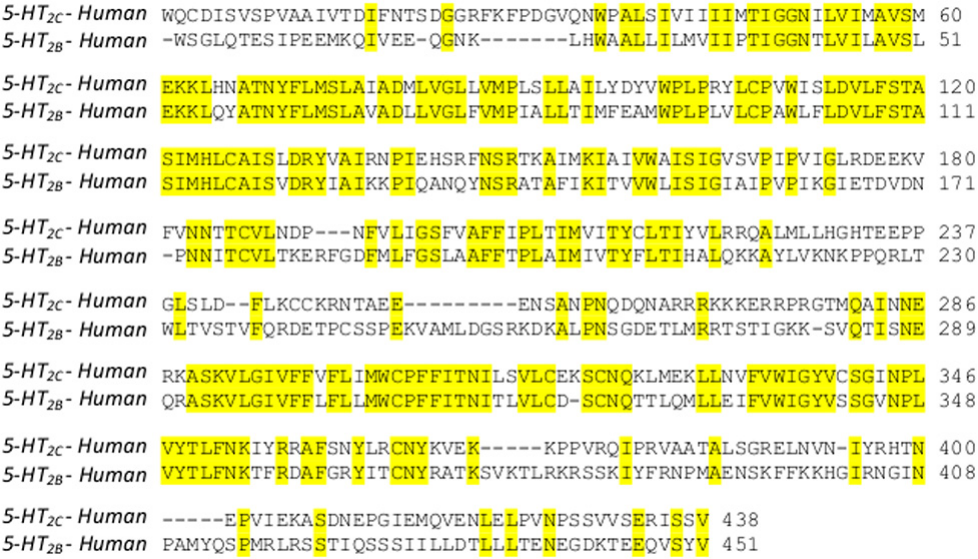
Alignment of 5-HT_2B_R and 5-HT_2C_R sequences using ClustalW. Conserved residues are highlighted in yellow.

The amino-acid sequence alignment of the α_7_, α_4_ and β_2_ subunits with their corresponding AChBP *and Torpedo* nAChR template subunits are shown in Figs 2 and 3, in which the conserved, semi-conserved residues and regions with conserved secondary structures are highlighted. Furthermore, the sequence alignment of the human 5-HT_2C_R with the 5-HT_2B_R used as template is shown in Fig 4.

As in previous work [20–23], we used these alignment data to generate the 3D models of the α7 nAChR (ECD and TMD) and the 5-HT_2C_R. The stereochemical and energetic quality of the resulting models were evaluated using ANOLEA server [24] and Procheck [25]. S1–S3 Figs show the Ramachandran plots for the ECD (S1 Fig) and the TMD (S2 Fig) models of the α7 nAChR, as well as for the 5-HT_2C_R (S3 Fig) model. As shown in the corresponding insets (S1–S3 Figs), in all cases more than 91% of residues are in the most favored regions, about 4–8% are in additional allowed regions and less than 3% are in generously allowed regions. No residues were found in disallowed regions, confirming the quality of the models.

### SB-206553 binding site location at 5-HT_2_ receptors

**Fig 5:**
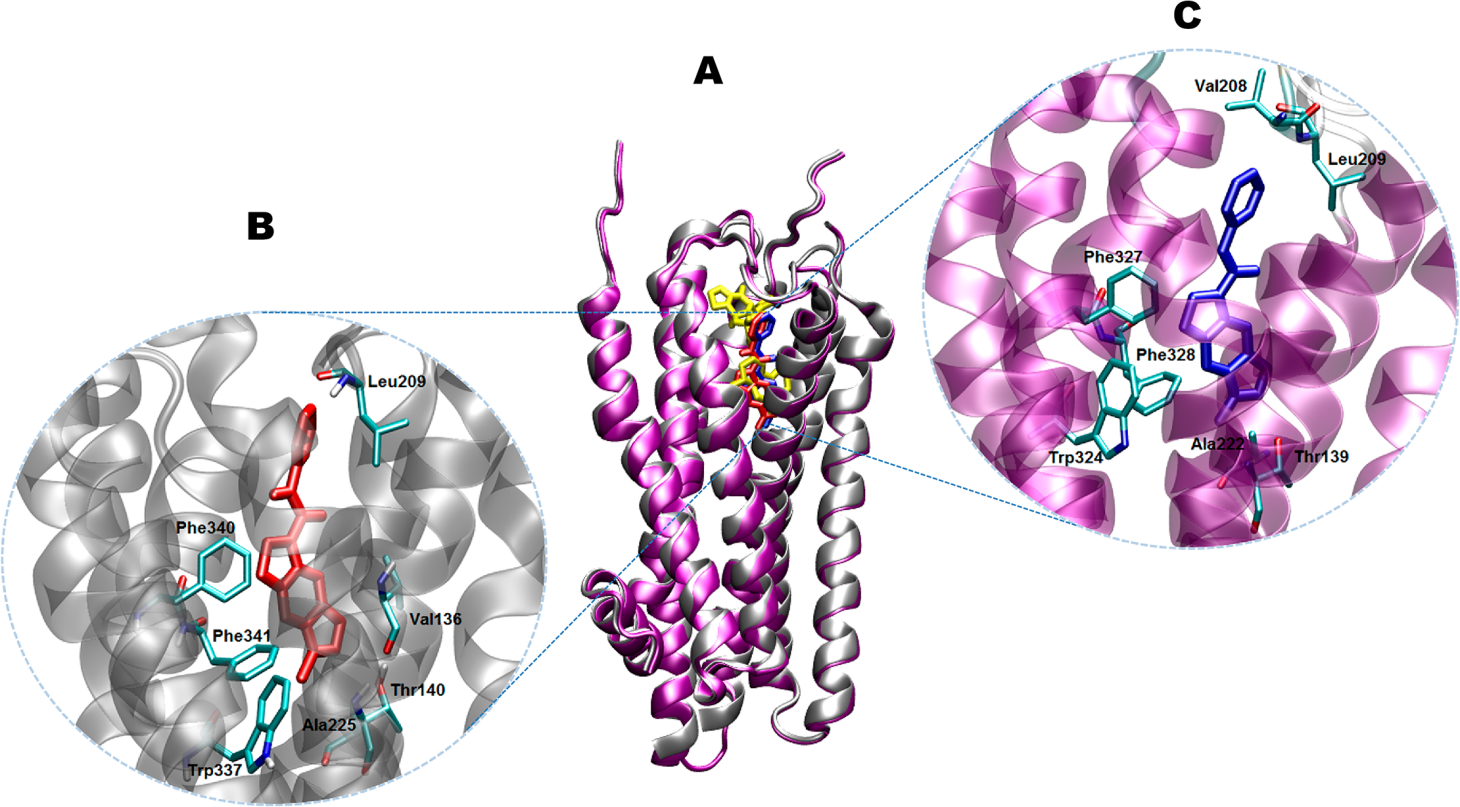
Structural determinants of the SB-206553 binding site at the 5-HT_2_Rs. (A) Ribbon diagram of the superimposed structures of the 5-HT_2B_R (silver) and 5-HT_2C_R (purple), showing the putative binding site for SB-206553 (red or blue, respectively) at each protein. For comparative purposes, the binding site for ergotamine (yellow) in the crystal structure of the 5-HT_2B_R (PDB code 4IB4) is also depicted. (B-C) Close ups of the docking poses of SB-206553 at 5-HT_2B_R and 5-HT_2C_R, respectively. Main active site amino acid residues (cyan) are rendered as stick models.

Fig 5A shows a general view for the most stable docking poses for SB-206553 when docked at either the 5-HT_2B_R or 5-HT_2C_R. The docking analysis of 150 simulations for each drug-receptor complex, revealed a total of five and seven multimember conformation clusters at the 5-HT_2B_R and 5-HT_2C_R, respectively (S4 and S5 Figs). In both cases, the configuration with the lowest binding energy (-17.2 kcal/mol and -15.3 kcal/mol, respectively) was a member of the highest populated cluster (S4 and S5 Figs). The models show that the drug docks in a very similar pose at both targets. Moreover, this binding site is located in a position analogous to that occupied by the agonist ergotamine in the crystal structure of the 5-HT_2B_R [12]. This result agrees with the competitive nature of the action of SB-206553 [7]. When these ligand-receptor complexes were submitted to 10-ns MD simulations, almost no changes were observed in the position adopted by SB-206553 at its binding sites (RMSD < 0.4 Å during the whole simulations). Furthermore, this site coincides with the binding pocket described for 5-HT and a series of 5-HT_2C_R antagonists and inverse agonists, as defined by a similar docking protocol as that used in the present work [33]. These observations support the plausibility of this location as the most probable binding site for SB-206553 at 5-HT_2_Rs.

As shown in the insets of Fig 5, the molecular modeling of the environment surrounding the binding poses of SB-206553 in the 5-HT_2B_R (Fig 5B) or 5-HT_2C_R (Fig 5C), suggests that the pyrroloindole moiety of this compound is located in a hydrophobic pocket formed by a series of aromatic and aliphatic residues embedded in the transmembrane domain, whereas its pyridine moiety is oriented towards the extracellular side of the receptor. In the particular case of the 5-HT_2B_R, the aromatic rings of Phe340 and Phe341 (homologous to Phe327 and Phe328 in the 5-HT_2C_R) are oriented in such a way that they establish π-π interactions with the ligand indole group. An additional interaction between the pyrroline moiety and Trp337 (Trp324 in the 5-HT_2C_R) is also observed. Furthermore, the centroid of the SB-206553 pyridine ring is located in a favorable position to establish van der Waals interactions with Leu209, which may further stabilize this binding mode. It should be noted that the aforementioned residues, particularly the aromatic ones, coincide with those underlying the binding of several 5-HT_2_ ligands, as identified in other docking [33,40–42] and site-directed mutagenesis [43–45] studies. Moreover, crystallographic data from 5-HT_2B_R indicate that a tightly bound water bridge is essential for stabilizing ergotamine at its binding site [12]. Considering the importance that water molecules in binding sites might have for intermolecular interactions and stability of different ligands [46,47], we investigated whether this may affect SB-206553 binding. Interestingly, our simulations showed that SB-206553 did not interact with the water molecule at any of the 5-HT_2_R receptors studied. This might be related with the smaller size of SB-206553 as compared with ergotamine and/or with their different intrinsic activities at these receptors (see below).

### SB-206553 binding site location in the α7 nAChR

**Fig 6:**
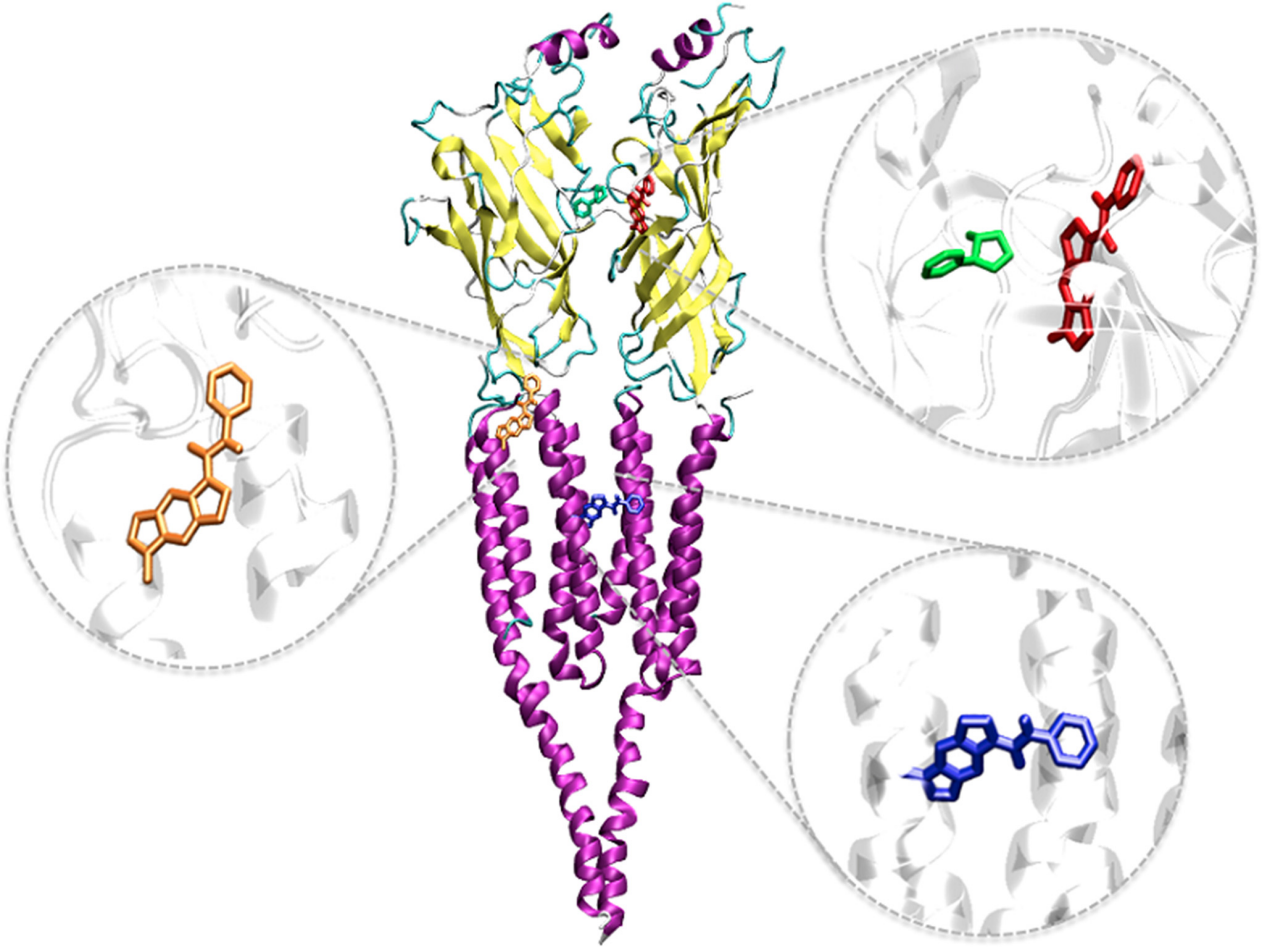
Potential binding sites for SB-206553 at the α7 nAChR. Ribbon diagram of the α7 nAChR model showing the putative binding sites for SB-206553 in the extracellular domain (ECD; red), at the M2-M3 loop (orange), and in the transmembrane domain (TMD; blue), respectively. β-Sheets and α-helices are shown in yellow and purple, respectively. The insets show molecular details of the docking poses of SB-206553 at each putative binding site. For comparative purposes, the binding pose of nicotine (green) in the crystal structure of the AChBP (PDB code 1UW6) is also depicted.

When SB-206553 was docked at both the ECD and TMD of the α7 nAChR, three possible binding sites were detected (Fig 6). The most stable poses (binding energy = -23.8 kcal/mol; S6 Fig) showed the compound docked in a pocket in the ECD, which is different from the orthosteric site occupied by nicotine. Nevertheless, similarly stable complexes (as judged by docking energies; S7 Fig) were detected with the drug bound to sites located in the M2-M3 loop and in the TMD of the α7 nAChR (Fig 6). Thus, after docking of SB-206553 at this region, six multimember conformation clusters were identified, with the two highest populated clusters having 72 (M2-M3 loop) and 68 (TMD) members out of 150 conformations (S7 Fig). The three sites identified have been previously shown to be the binding site (or to modulate the effects) of different positive allosteric modulators (PAMs) [48–53], and therefore any of them might account for the pharmacological effects of SB-206553 at this receptor. Since none of these binding sites could be *a priori* selected/discarded based on energy criteria or previous data, MD simulations were performed to evaluate the stability of the ligand at each one of the potential binding sites.

**Fig 7:**
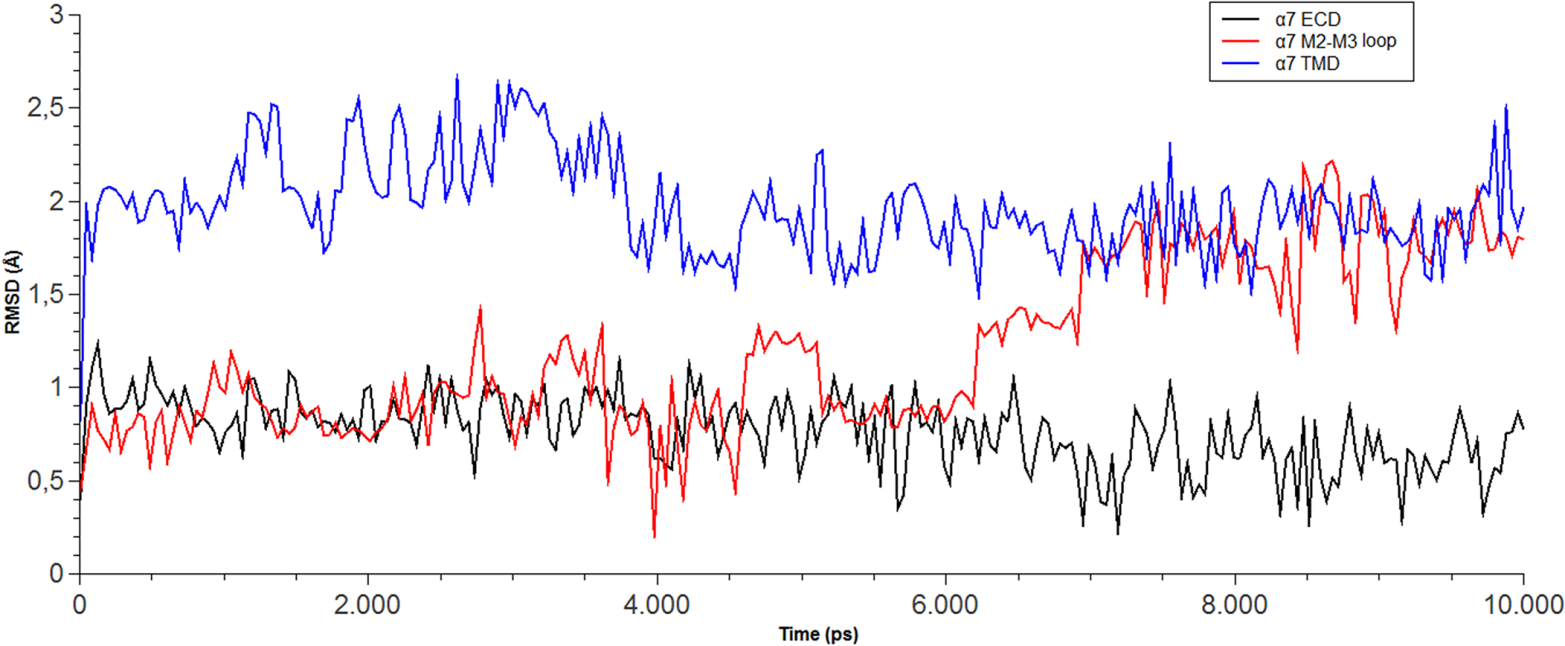
RMSD behavior of SB-206553 docked in each one of the three putative binding sites from the α7 nAChR. RMSD values are shown for the compound when bound at: the extracellular domain (ECD; black line), the M2-M3 loop (red line), and the transmembrane domain (TMD; blue line).

Fig 7 shows the movement of SB-206553 during the 10-ns MD simulation, at each one of the potential binding sites of the α7 nAChR. In the site located in the α7 nAChR-ECD, SB-206553 remained relatively stable (in the same position) during the whole MD simulation. Conversely, the complexes were clearly less stable in the cases in which the drug was bound to either the M2-M3 region or the TMD. These results suggest that the most likely binding site for SB-206553 at the α7 nAChR is the allosteric site located in the ECD. It should be noted that this site is located in a position that roughly coincides with the “vestibule pocket”, an allosteric site identified in a recent crystallographic study that used a chimera of the α7 nAChR and AChBP as a model for the ECD of the α7 nAChR [54].

**Fig 8:**
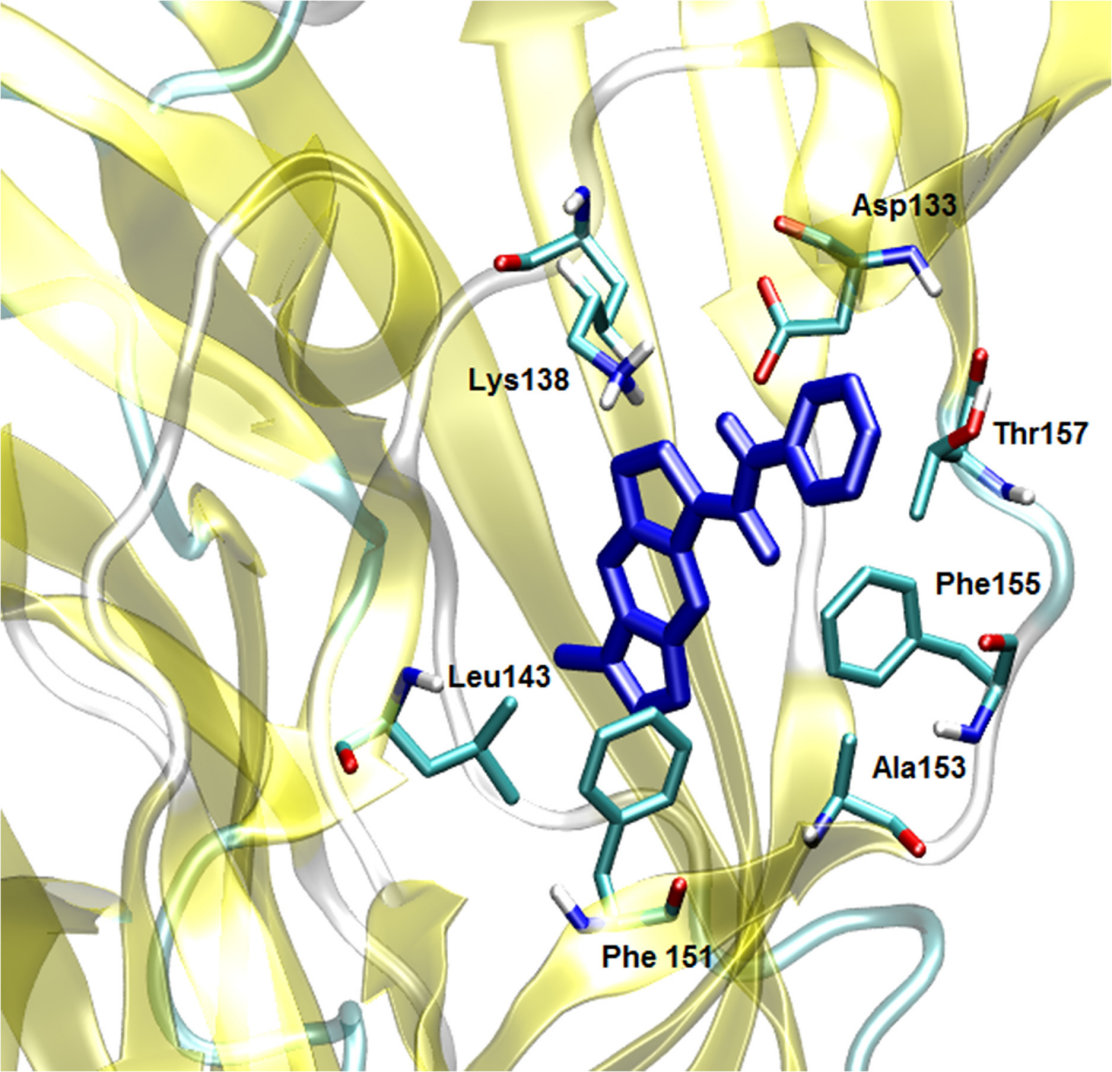
Binding mode of SB-206553 at the extracellular domain of the α7 nAChR. SB-206553 is shown in blue. Main active site amino acid residues (cyan) are rendered as stick models.

As seen in Fig 8, SB-206553 exhibits a binding mode in the α7 nAChR-ECD where the amino group of the carbamate establishes a hydrogen bond with the carbonyl group of Asp133, whereas the pyrroloindole moiety appears located between Phe151 and Phe155, a position that would favor π-stacking interactions between the corresponding aromatic rings. In addition, hydrophobic interactions with Lys138, Leu143, Ala153 and Thr157 were observed, all of which could further stabilize the binding mode of SB-206553 at this site.

### Similarities between the binding sites of SB-206553 at the 5-HT_2_ and α7 nACh receptors

As mentioned in the Methods section, to assess the structural similarity between all binding sites, the residues located at a given distance of 3.5 to 8.0 Å from the docked ligand were considered.

**Fig 9:**
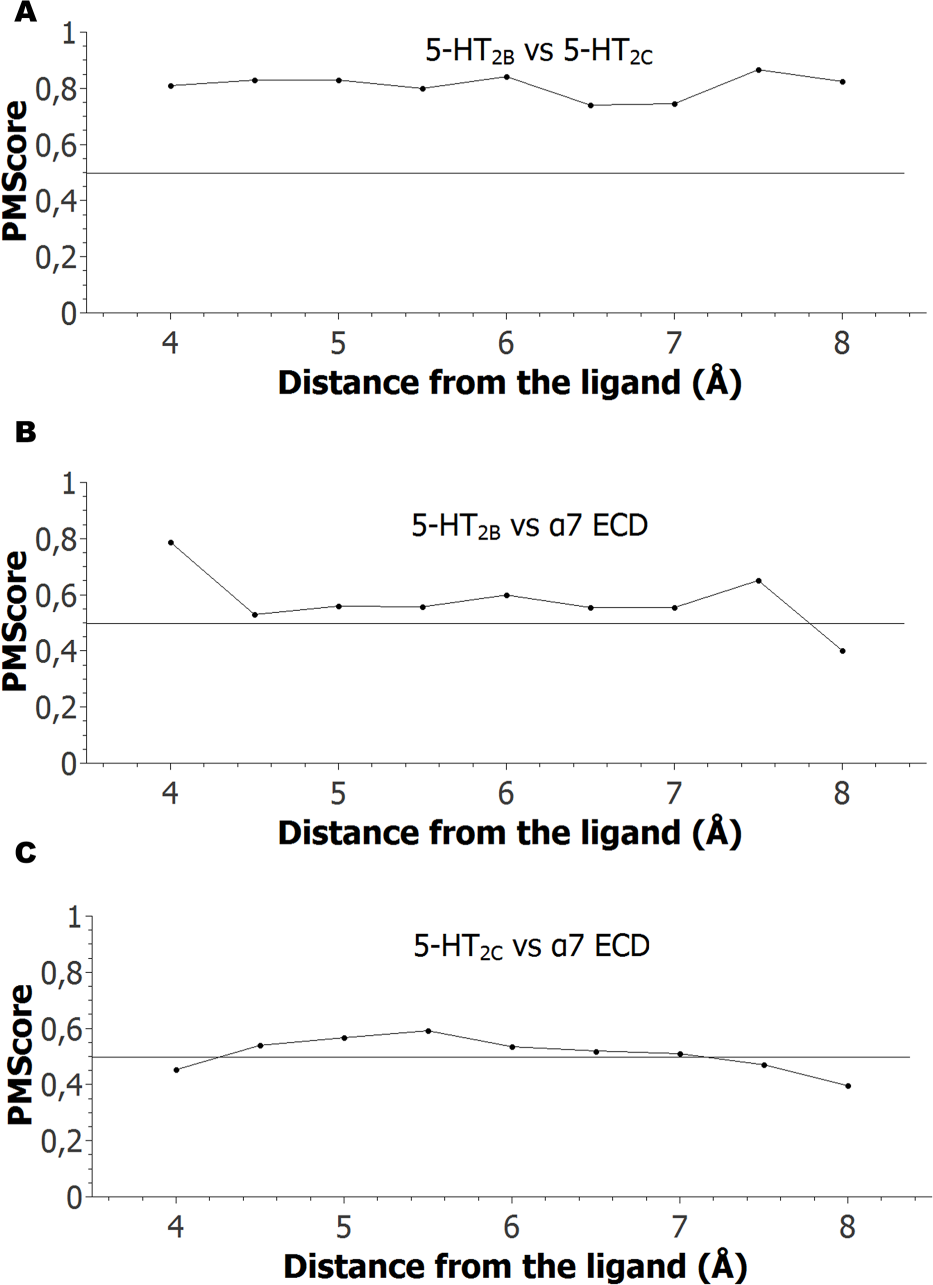
Similarity profiles between the binding sites for SB-206553 at the 5-HT_2B_R, 5-HT_2C_R, and α7 nAChR. Similarity profiles between the binding sites for SB-206553 docked at the 5-HT_2B_R and 5-HT_2C_ R (A), at the 5-HT_2B_R and α7 nAChR (B), and at the 5-HT_2B_R and α7 nAChR (C), as calculated using PocketMatch. In each case, the horizontal black line indicates PMScore = 0.5. Each point corresponds to the PMScore.

Fig 9 shows the similarity profile, i.e. the PMScores determined at different distances from the ligand (3.5 Å to 8.0 Å), for the binding sites of SB-206553 at 5-HT_2B_, 5-HT_2C_ and α7-nACh receptors. When comparing the 5-HT_2B_R and 5-HT_2C_R binding sites (Fig 9A), the calculated similarity scores at all measured distances were well above 0.5, indicating high similarity, as expected for two highly homologous proteins triggered by the same neurotransmitter. Since most amino acids located between 4 and 6 Å from the ligand line the binding site (see Fig 5B and 5C), this result agrees with the idea that the binding sites at the 5-HT_2B_R and 5-HT_2C_R are quite similar. In addition, our data show that the similarity extends beyond the binding site, with both proteins showing a high degree of global structural similarity, considering at least all the residues located up to 8 Å from the ligand. Even though these findings might be considered obvious from the analysis of each receptor sequence and function, they confirm the suitability of PocketMatch to find and predict such characteristics. They also highlight how challenging it is to design compounds that can distinguish between receptors belonging to the 5-HT_2_R family [55].

On the other hand, when the binding site of SB-206553 at the α7 nAChR-ECD was compared to those at the 5-HT_2B_R (Fig 9B) and 5-HT_2C_R (Fig 9C), PMScore values above 0.5 were observed at least from 4.5 to 7.0 Å from the ligand. This indicates that these binding sites are similar, both in size and chemical nature of the amino acid residues lining these pockets. In contrast to that observed for 5-HT_2_Rs, the detected similarity does not extend beyond the binding sites. This is sound in view of the high structural and functional diversity of these receptors. This result is remarkable since it shows that two profoundly different targets, such as an ionotropic nAChR and metabotropic 5-HT_2_Rs, share a similar pocket, which is also pharmacologically relevant. In addition, our results provide a rationale to explain the affinity showed by SB-206553 upon these two highly different types of proteins.

Interestingly, when the putative binding sites for SB-206553 located in the M2-M3 loop and in the TMD of the α7 nAChR were evaluated against the 5-HT_2_Rs binding sites, no similarity was detected (all PMScore < 0.5) at any distance measured (S8 and S9 Figs). This result further supports our initial proposal that the active site for SB-206553 is located in the α7 nAChR-ECD.

### Characteristics of the common binding site for SB-206553 at the 5-HT_2_ and α7 nACh receptors

**Fig 10:**
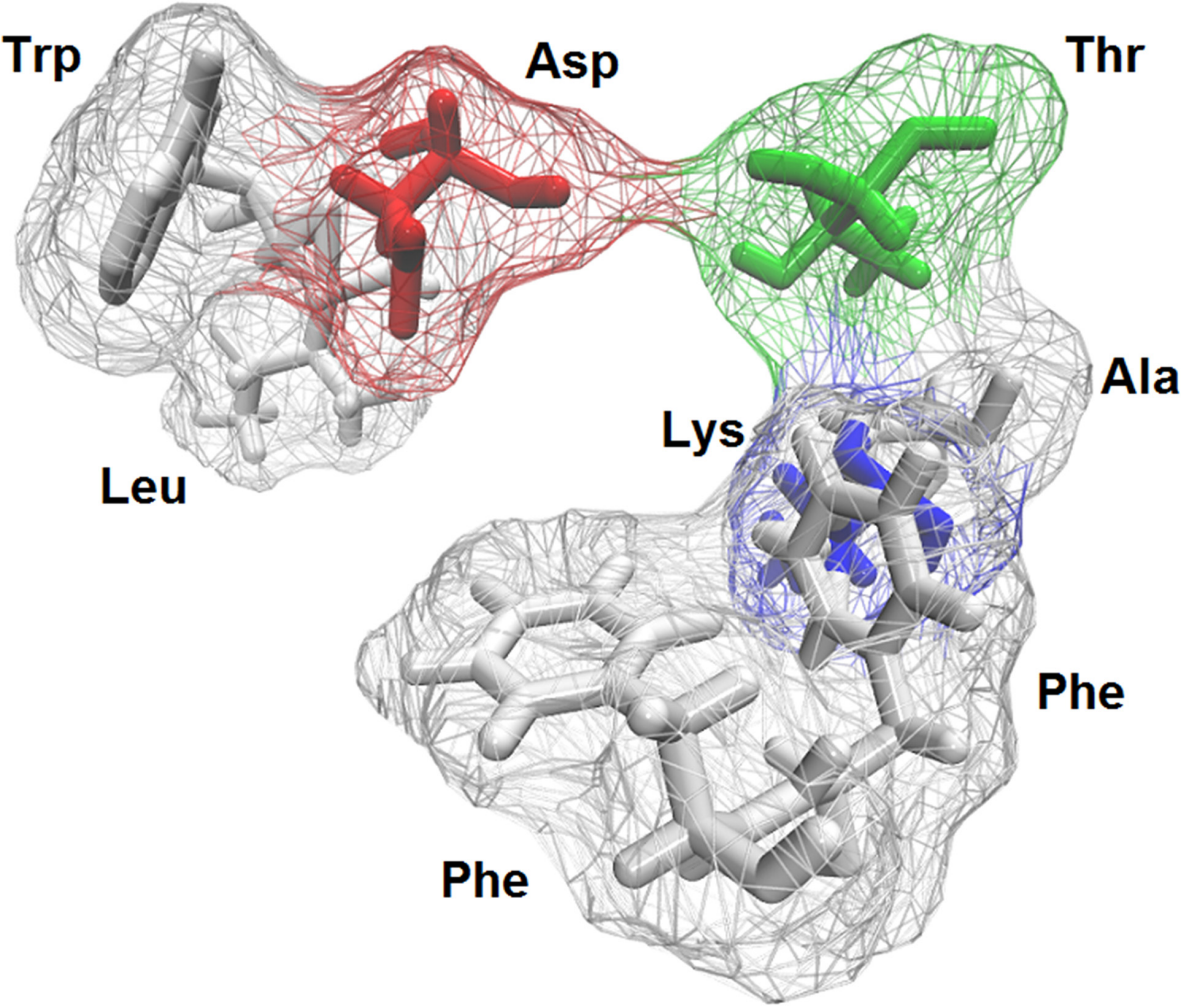
Common structure of the SB-206553 binding site of the 5-HT_2_Rs and α7 nAChR. The cavity observed in the site is depicted as a transparent surface with residues in licorice format. Each color represents the chemical nature of residues (polar = green, non-polar = grey, negatively charged = red, positively charged = blue).

After the alignment of the SB-206553 binding sites at the 5-HT_2_Rs and α7 nAChR, a consensus binding site was generated (Fig 10). This three-dimensional pattern contains the residues which are likely responsible for the binding of the drug to the different targets. The consensus binding site is a hydrophobic pocket in which three aromatic residues play a crucial role to establish π-π and/or hydrophobic interactions with the ligand’s indole group, in agreement with our previous simulation. The importance of aromatic interactions for the binding of different ligands at 5-HT_2_Rs has been documented before [33,43–45]. Importantly, our results show that a similar three-dimensional arrangement is also present in an allosteric site in the α7 nAChR.

In addition, the consensus binding site includes a Leu, a Thr, and an Ala residue, which may help to stabilize the ligand binding (e.g., *via* hydrophobic interactions). It is noteworthy that our analysis indicates that the consensus binding site also contains an aspartic acid. In the docking studies, an interaction between SB-206553 and an Asp residue was only observed in the α7 nAChR. However, an important interaction between different agonists and Asp134 at the orthosteric binding sites of 5-HT_2_Rs has been previously reported (see e.g., [56]). The fact that this type of interaction was not detected in our docking simulations might be related to the inverse agonist properties of SB-206553.

Beyond these considerations, the consensus binding site determined in this work should be useful for the rational design of novel compounds acting simultaneously at 5-HT_2_ and α7 nACh receptors.

## Conclusions

SB-206553 is a multitarget drug with an interesting pharmacological profile ranging from anxiolytic to anti-addictive properties [9,57]. It is likely that these actions are mediated by its inverse agonist activity at 5-HT_2B/2C_Rs and its positive allosteric modulatory activity at α7 nAChR.

In this work we demonstrated that both kinds of receptor targets have similar binding sites, which presumably underlies the polypharmacological properties mediated by SB-206553. The identification of this common binding site should facilitate the structure-based design of novel drugs acting at both receptor types which, depending on their intrinsic activities (agonist, antagonist, positive or negative modulator, etc.), might exhibit a wide spectrum of pharmacological actions.

On the other hand, our results give further support to the notion that all proteins targeted by a given compound may have certain similarities at their binding sites, and therefore searching for these similarities is a key aspect in the rational design of polypharmacological drugs.

## Supporting Information

S1 Fig Ramachandran plot for the homology model generated for the extracellular domain (ECD) of the α7 nAChR. Inset shows Procheck statistics for the model.
(TIF)

S2 Fig Ramachandran plot for the homology model generated for the transmembrane domain (TMD) of the α7 nAChR. Inset shows Procheck statistics for the model.
(TIF)

S3 Fig Ramachandran plot for the homology model generated for the 5-HT_2C_R. Inset shows Procheck statistics for the model.
(TIF)

S4 Fig Docking of SB-206553 at the 5-HT_2B_R. Figure shows the cluster analyses of the AutoDock docking runs of SB-206553 in the drug binding site at the 5-HT_2B_R.
(TIF)

S5 Fig Docking of SB-206553 at the 5-HT_2C_R. Figure shows the cluster analyses of the AutoDock docking runs of SB-206553 in the drug binding site at the 5-HT_2C_R.
(TIF)

S6 Fig Docking of SB-206553 at the ECD of the α7 nAChR. Figure shows the cluster analyses of the AutoDock docking runs of SB-206553 in the drug binding site at the ECD of the α7 nAChR.
(TIF)

S7 Fig Docking of SB-206553 at the TMD of the α7 nAChR. Figure shows the cluster analyses of the AutoDock docking runs of SB-206553 in the drug binding site at the TMD of the α7 nAChR.
(TIF)

S8 Fig Similarity profiles of the binding sites for SB-206553. Similarity profiles between the binding sites of SB-206553 docked in the 5-HT_2B_R and the transmembrane domain (TMD) of the α7 nAChR (A), in the 5-HT_2B_R and the M2-M3 loop from the α7 nAChR (B), as calculated using PocketMatch. In each case, the horizontal black line indicates PMScore = 0.5. Each point corresponds to the PMScore.
(TIF)

S9 Fig Similarity profiles of the binding sites for SB-206553. Similarity profiles between the binding sites for SB-206553 docked in the 5-HT_2C_R and the transmembrane domain (TMD) of the α7 nAChR (A), and in the 5-HT_2C_R and the M2-M3 loop from the α7 nAChR (B), as calculated using PocketMatch. In each case, the horizontal black line indicates PMScore = 0.5. Each point corresponds to the PMScore.
(TIF)

## References

[1] Hopkins AL. Network pharmacology: the next paradigm in drug discovery. Nat Chem Biol. 2008;4: 682‐690. 10.1038/nchembio.118 18936753

[2] Schrattenholz A, Soskić V. What does systems biology mean for drug development? Curr Med Chem. 2008;15: 1520–1528. 18537627 10.2174/092986708784638843

[3] Zhao S, Iyengar R. Systems pharmacology: network analysis to identify multiscale mechanisms of drug action. Annu Rev Pharmacol Toxicol. 2012;52: 505‐521. 10.1146/annurev-pharmtox-010611-134520 22235860 PMC3619403

[4] Anighoro A, Bajorath J, Rastelli G. Polypharmacology: challenges and opportunities in drug discovery. J Med Chem. 2014;19: 7874–7887. 10.1021/jm5006463 24946140

[5] Rognan D. Structure-based approaches to target fishing and ligand profiling. Mol Inf. 2010;29: 176–187. 10.1002/minf.200900081 27462761

[6] Kahraman A, Morris RJ, Laskowski RA, Thornton JM. Shape variation in protein binding pockets and their ligands. J Mol Biol. 2007;368: 283–301. 17337005 10.1016/j.jmb.2007.01.086

[7] Forbes IT, Ham P, Booth DH, Martin RT, Thompson M, Baxter GS, et al 5-Methyl-1-(3-pyridylcarbamoyl)-1,2,3,5-tetrahydropyrrolo[2,3-f]indole: a novel 5-HT2C/5-HT2B receptor antagonist with improved affinity, selectivity, and oral activity. J Med Chem. 1995;38: 2524–2530. 7629791 10.1021/jm00014a004

[8] Kennett GA, Wood MD, Bright F, Cilia J, Piper DC, Gager T, et al In vitro and in vivo profile of SB 206553, a potent 5-HT2C/5-HT2B receptor antagonist with anxiolytic-like properties. Br J Pharmacol. 1996;117: 427–434. 8821530 10.1111/j.1476-5381.1996.tb15208.x PMC1909304

[9] Berg KA, Stout BD, Cropper JD, Maayani S, Clarke WP. Novel actions of inverse agonists on 5-HT2C receptor systems. Mol Pharmacol. 1999;55: 863–872. 10220565

[10] Dunlop J, Lock T, Jow B, Sitzia F, Grauer S, Jow F, et al Old and new pharmacology: positive allosteric modulation of the alpha7 nicotinic acetylcholine receptor by the 5-hydroxytryptamine (2B/C) receptor antagonist SB-206553 (3,5-dihydro-5-methyl-N-3-pyridinylbenzo[1,2-b:4,5-b']di pyrrole-1(2H)-carboxamide. J Pharmacol Exp Ther. 2009;3: 766–776. 10.1124/jpet.108.146514 19050173

[11] Bockaert J, Claeysen S, Becamel C, Dumuis A, Marin P. Neuronal 5-HT metabotropic receptors: fine-tuning of their structure, signaling, and roles in synaptic modulation. Cell Tissue Res. 2006;326: 553–572. 16896947 10.1007/s00441-006-0286-1

[12] Wacker D, Wang C, Katritch V, Han GW, Huang XP, Vardy E, et al Structural features for functional selectivity at serotonin receptors. Science. 2013;340: 615–619. 10.1126/science.1232808 23519215 PMC3644390

[13] Papke RL. Merging old and new perspectives on nicotinic acetylcholine receptors. Biochem Pharmacol. 2014;89: 1–11. 10.1016/j.bcp.2014.01.029 24486571 PMC4755309

[14] Grutter T, Le Novère N, Changeux JP. Rational understanding of nicotinic receptors drug binding. Curr Top Med Chem. 2004;4: 645–650. 14965300 10.2174/1568026043451177

[15] Sali A, Blundell TL. Comparative protein modelling by satisfaction of spatial restraints. J Mol Biol. 1993;234: 779–815. 8254673 10.1006/jmbi.1993.1626

[16] Celie PH, van Rossum-Fikkert SE, van Dijk WJ, Brejc K, Smit AB, Sixma TK. Nicotine and carbamylcholine binding to nicotinic acetylcholine receptors as studied in AChBP crystal structures. Neuron. 2004;41: 907–914. 15046723 10.1016/s0896-6273(04)00115-1

[17] Unwin N. Refined structure of the nicotinic acetylcholine receptor at 4 angstrom resolution. J Mol Biol. 2005;346: 967–989. 15701510 10.1016/j.jmb.2004.12.031

[18] Thompson JD, Higgins DG, Gibson TJ. Clustalw: improving the sensitivity of progressive multiple sequence alignment through sequence weighting, position-specific gap penalties and weight matrix choice. Nucleic Acids Res. 1994;22: 4673–4680. 7984417 10.1093/nar/22.22.4673 PMC308517

[19] Abagyan R, Totrov M, Kuznetsov D. ICM-A new method for protein modeling and design: Applications to docking and structure prediction from the distorted native conformation. J Comput Chem. 1994;15: 488–506.

[20] Iturriaga-Vásquez P, Carbone A, García-Beltrán O, Livingstone PD, Biggin PC, Cassels BK, et al Molecular determinants for competitive inhibition of alpha α4β2 nicotinic acetylcholine receptors. Mol Pharmacol. 2010;78: 366–375. 10.1124/mol.110.065490 20547737 PMC2939478

[21] Faundez-Parraguez M, Farias-Rabelo N, Gonzalez-Gutierrez JP, Etcheverry-Berrios A, Alzate-Morales J, Adasme-Carreño F, et al Neonicotinic analogues: selective antagonists for α4β2 nicotinic acetylcholine receptors. Bioorg Med Chem. 2013;21: 2687–2694. 10.1016/j.bmc.2013.03.024 23561269

[22] Pessoa-Mahana H, González-Lira C, Fierro A, Zapata-Torres G, Pessoa-Mahana CD, Ortiz-Severin J, et al Synthesis, docking and pharmacological evaluation of novel homo- and hetero-bis 3-piperazinylpropylindole derivatives at SERT and 5-HT1A receptor. Bioorg Med Chem. 2013;21: 7604–7611. 10.1016/j.bmc.2013.10.036 24262884

[23] Pessoa-Mahana H, Núñez CU, Araya-Maturana R, Barría CS, Zapata-Torres G, Pessoa-Mahana CD, et al Synthesis, 5-hydroxytryptamine1A receptor affinity and docking studies of 3-[3-(4-aryl-1-piperazinyl)-propyl]-1H-indole derivatives. Chem Pharm Bull. 2012;60: 632–638. 22689401 10.1248/cpb.60.632

[24] Melo F, Devos D, Depiereux E, Feytmans E. ANOLEA: a www server to assess protein structures. Proc Int Conf Intell Syst Mol Biol. 1997;5: 187–190. 9322034

[25] Laskowski RA, MacArthur MW, Moss DS, Thornton JM. PROCHECK: a program to check the stereochemical quality of protein structures. J Appl Crystallogr. 1993;26: 283–291.

[26] Schrödinger Suite 2009 Protein Preparation Wizard, Schrödinger, LLC, New York, NY, 2011.

[27] Arias HR, Gu RX, Feuerbach D, Guo BB, Ye Y, Wei DQ. Novel positive allosteric modulators of the human α7 nicotinic acetylcholine receptor. Biochemistry. 2011;50: 5263–5278. 10.1021/bi102001m 21510634

[28] Maestro-Desmond Interoperability Tools, version 3.0, Schrödinger, New York, NY, 2011.

[29] National Center for Biotechnology Information. PubChem Compound Database; CID = 5163. Available: http://pubchem.ncbi.nlm.nih.gov/compound/5163. Accessed 1 Aug 2014).

[30] Stewart JJP. MOPAC: A general molecular orbital package. Quant Chem Prog Exch. 1990;10: 86.

[31] Morris M, Goodsell S, Halliday S, Huey R, Hart E, Belew K, et al Automated docking using a lamarckian genetic algorithm and an empirical binding free energy function. J Comput Chem. 1998;19: 1639–1662.

[32] Sotomayor-Zárate R, Quiroz G, Araya KA, Abarca J, Ibáñez MR, Montecinos A, et al 4-Methylthioamphetamine increases dopamine in the rat striatum and has rewarding effects in vivo. Basic Clin Pharmacol Toxicol. 2012;111: 371–379. 10.1111/j.1742-7843.2012.00926.x 22788961

[33] Galeazzi R, Massaccesi L, Piva F, Principato G, Laudadio E. Insights into the influence of 5-HT2c aminoacidic variants with the inhibitory action of serotonin inverse agonists and antagonists. J Mol Model. 2014;20: 2120 10.1007/s00894-014-2120-0 24562856

[34] Alzate-Morales JH, Vergara-Jaque A, Caballero J. Computational study on the interaction of N1 substituted pyrazole derivatives with B-Raf Kinase: an unusual water wire hydrogenbond network and novel interactions at the entrance of the active site. J Chem Inf Model. 2010;50: 1101–1112. 10.1021/ci100049h 20524689

[35] Stone J, Cohen J, Sotomayor M, Villa E. VMD Molecular Graphics tutorial. 2007.

[36] Yeturu K, Chandra N. PocketMatch: a new algorithm to compare binding sites in protein structures. BMC Bioinformatics. 2008;9: 543 10.1186/1471-2105-9-543 19091072 PMC2639437

[37] Fierro A, Montecinos A, Gómez-Molina C, Núñez G, Aldeco M, Edmondson DE, et al Similarities between the binding sites of monoamine oxidase (MAO) from different species. Is zebrafish a useful model for the discovery of novel MAO inhibitors? In: Baptista GR, Editor. An integrated view of the molecular recognition and toxinology. From analytical procedures to biomedical applications. Rijeka: InTech—Open Access Publisher; 2013 pp. 405–431.

[38] Shulman-Peleg A, Shatsky M, Nussinov R, Wolfson HJ. MultiBind and MAPPIS: webservers for multiple alignment of protein 3D-binding sites and their interactions. Nucleic Acids Res. 2008;36 (Web Server issue): W260–264. 10.1093/nar/gkn185 18467424 PMC2447750

[39] Wolfson HJ. Model-based object recognition by geometric hashing In: Faugeras O, Editor. Computer vision ECCV90. Heidelberg: Springer, 1990 pp. 526–536.

[40] Cordova-Sintjago T, Sakhuja R, Kondabolu K, Canal CE, Booth RG. Molecular determinants for ligand binding at serotonin 5-HT2A and 5-HT2C GPCRs: experimental affinity results analyzed by molecular modeling and ligand docking studies. Int J Quantum Chem. 2012;112: 3807–3814. 23913978 10.1002/qua.24237 PMC3729958

[41] Kim SK, Li Y, Abrol R, Heo J, Goddard WA III. Predicted structures and dynamics for agonists and antagonists bound to serotonin 5-HT2B and 5-HT2C receptors. J Chem Inf Model. 2011;51: 420–433. 10.1021/ci100375b 21299232 PMC3070210

[42] Lu C, Jin F, Li C, Li W, Liu G, Tang Y. Insights into binding modes of 5-HT2c receptor antagonists with ligand-based and receptor-based methods. J Mol Model. 2011;17: 2513–2523. 10.1007/s00894-010-0936-9 21203788

[43] Cordova-Sintjago TC, Villa N, Fang L, Booth RG. Aromatic interactions impact ligand binding and function at serotonin 5-HT2C G protein-coupled receptors: receptor homology modelling, ligand docking, and molecular dynamics results validated by experimental studies. Mol Phys. 2014;112: 398–407. 24729635 10.1080/00268976.2013.833656 PMC3979624

[44] Braden MR, Parrish JC, Naylor JC, Nichols DE. Molecular interaction of serotonin 5-HT2A receptor residues Phe339(6.51) and Phe340(6.52) with superpotent N-benzyl phenethylamine agonists. Mol Pharmacol. 2006;70: 1956–1964. 17000863 10.1124/mol.106.028720

[45] Roth BL, Shoham M, Choudhary MS, Khan N. Identification of conserved aromatic residues essential for agonist binding and second messenger production at 5-hydroxytryptamine2A receptors. Mol Pharmacol. 1997;52: 259–266. 9271348 10.1124/mol.52.2.259

[46] García-Sosa AT. Hydration properties of ligands and drugs in protein binding sites: tightly-bound, bridging water molecules and their effects and consequences on molecular design strategies. J Chem Inf Model. 2013;53: 1388–1405. 10.1021/ci3005786 23662606

[47] Bissantz C, Kuhn B, Stahl M. A medicinal chemist's guide to molecular interactions. J Med Chem. 2010;53: 5061–5084. 10.1021/jm100112j 20345171 PMC2905122

[48] Gill JK, Savolainen M, Young GT, Zwart R, Sher E, Millar NS. Agonist activation of alpha7 nicotinic acetylcholine receptors via an allosteric transmembrane site. Proc Natl Acad Sci USA. 2011;108: 5867–5872. 10.1073/pnas.1017975108 21436053 PMC3078348

[49] Collins T, Young GT, Millar NS. Competitive binding at a nicotinic receptor transmembrane site of two α7-selective positive allosteric modulators with differing effects on agonist-evoked desensitization. Neuropharmacology. 2011;61: 1306–1313. 10.1016/j.neuropharm.2011.07.035 21820451 PMC3205184

[50] Castillo M, Mulet J, Bernal JA, Criado M, Sala F, Sala S. Improved gating of a chimeric alpha7-5HT3A receptor upon mutations at the M2-M3 extracellular loop. FEBS Lett. 2006;580: 256–260. 16364316 10.1016/j.febslet.2005.12.010

[51] Iorga B, Herlem D, Barré E, Guillou C. Acetylcholine nicotinic receptors: finding the putative binding site of allosteric modulators using the "blind docking" approach. J Mol Model. 2006;12: 366–372. 16372175 10.1007/s00894-005-0057-z

[52] Young GT, Zwart R, Walker AS, Sher E, Millar NS. Potentiation of alpha7 nicotinic acetylcholine receptors via an allosteric transmembrane site. Proc Natl Acad Sci USA. 2008;105: 14686–14691. 10.1073/pnas.0804372105 18791069 PMC2535569

[53] Bertrand D, Bertrand S, Cassar S, Gubbins E, Li J, Gopalakrishnan M. Positive allosteric modulation of the alpha7 nicotinic acetylcholine receptor: ligand interactions with distinct binding sites and evidence for a prominent role of the M2-M3 segment. Mol Pharmacol. 2008;74: 1407–1416. 10.1124/mol.107.042820 18678621

[54] Spurny R, Debaveye S, Farinha A, Veys K, Vos AM, Gossas T, Atack J, Bertrand S, Bertrand D, Danielson UH, Tresadern G, Ulens C. Molecular blueprint of allosteric binding sites in a homologue of the agonist-binding domain of the α7 nicotinic acetylcholine receptor. Proc Natl Acad Sci U S A. 2015;112: E2543–2552. 10.1073/pnas.1418289112 25918415 PMC4434711

[55] Knight AR, Misra A, Quirk K, Benwell K, Revell D, Kennett G, et al Pharmacological characterisation of the agonist radioligand binding site of 5-HT(2A), 5-HT(2B) and 5-HT(2C) receptors. Naunyn Schmiedebergs Arch Pharmacol. 2004;370: 114–123. 15322733 10.1007/s00210-004-0951-4

[56] Isberg V, Paine J, Leth-Petersen S, Kristensen JL, Gloriam DE. Structure-activity relationships of constrained phenylethylamine ligands for the serotonin 5-HT2 receptors. PLoS One 2013;8: e78515 10.1371/journal.pone.0078515 24244317 PMC3820707

[57] Graves SM, Napier TC. SB 206553, a putative 5-HT2C inverse agonist, attenuates methamphetamine-seeking in rats. BMC Neurosci. 2012;13: 65 10.1186/1471-2202-13-65 22697313 PMC3441362

