# Supplementary figures and images for: Similarities between the Binding Sites of SB-206553 at Serotonin Type 2 and Alpha7 Acetylcholine Nicotinic Receptors: Rationale for Its Polypharmacological Profile
Source: PLoS One. 2015 Aug 5;10(8):e0134444. doi: 10.1371/journal.pone.0134444 (PMC4526571; doi:10.1371/journal.pone.0134444)

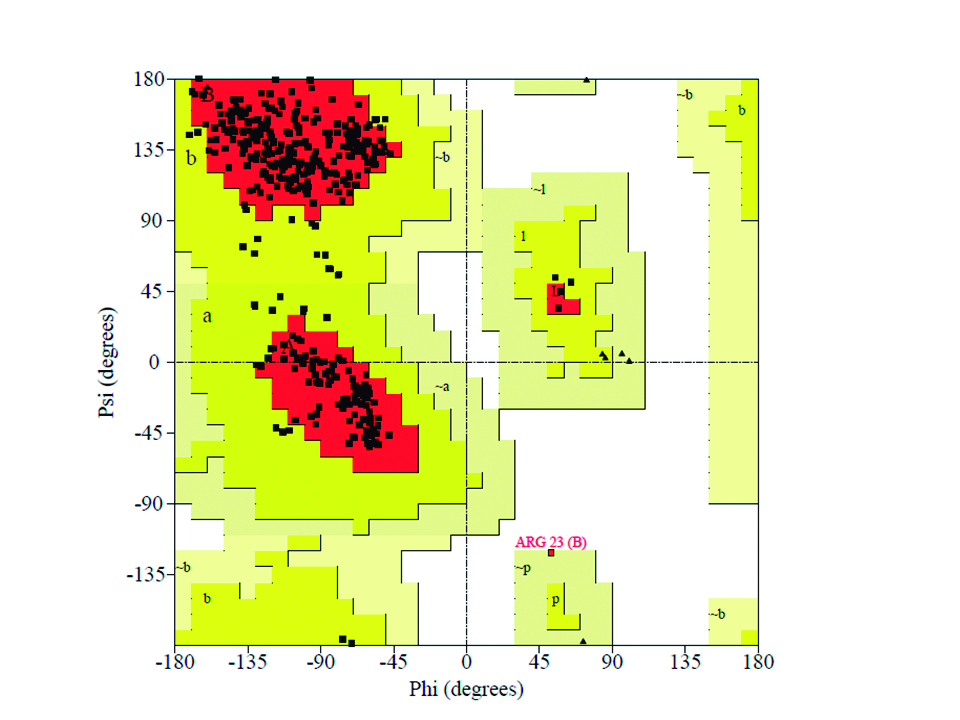

Supplement: S1 Fig — Inset shows Procheck statistics for the model. (TIF) [file pone.0134444.s001.tif]

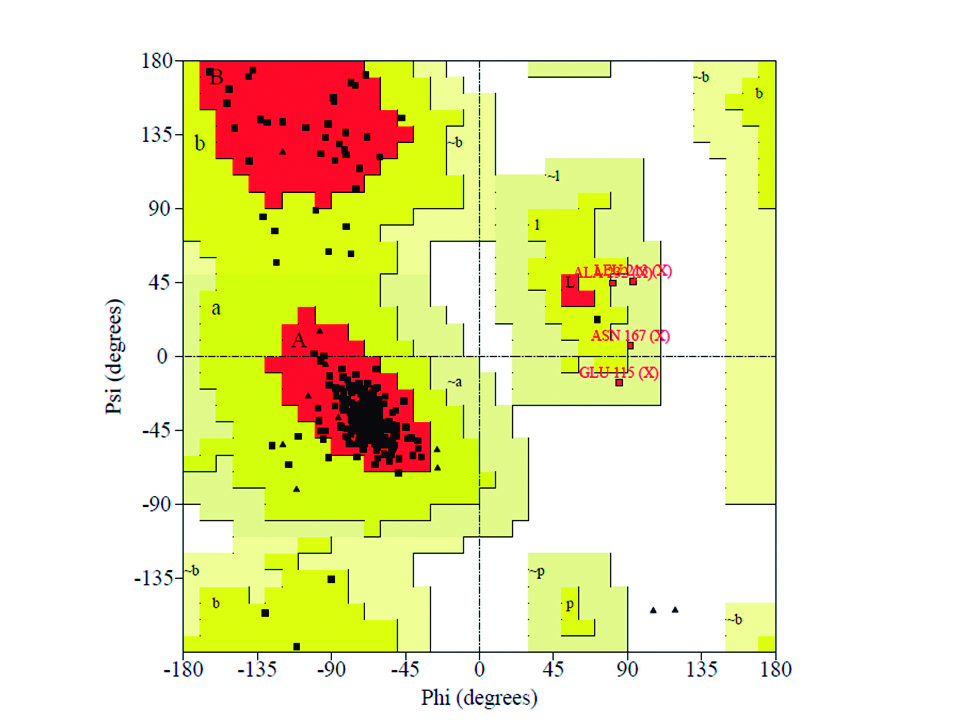

Supplement: S2 Fig — Inset shows Procheck statistics for the model. (TIF) [file pone.0134444.s002.tif]

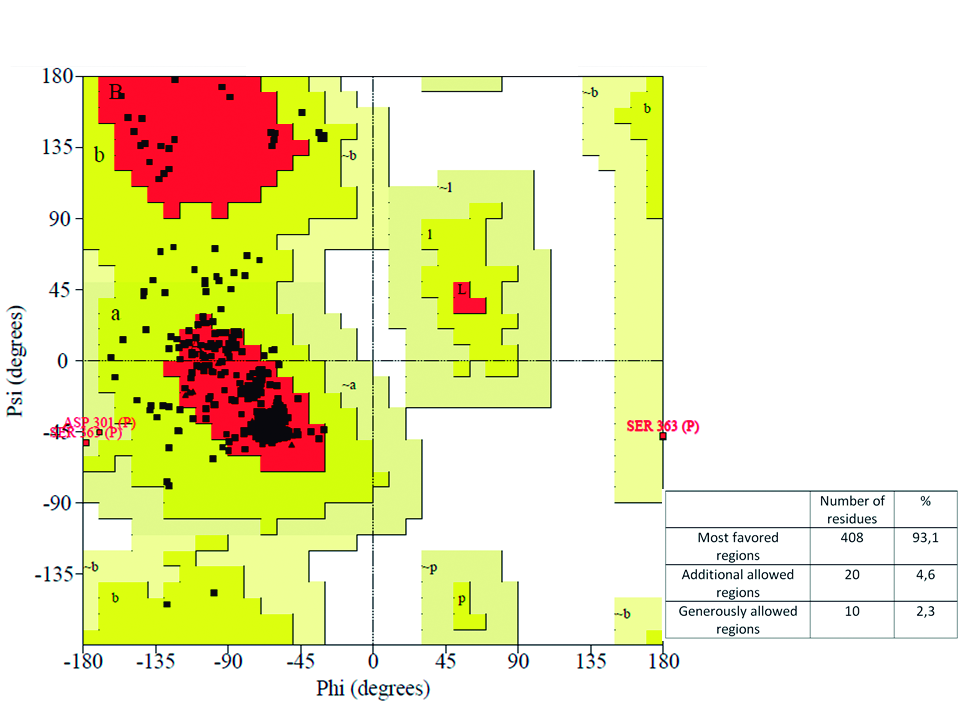

Supplement: S3 Fig — Inset shows Procheck statistics for the model. (TIF) [file pone.0134444.s003.tif]

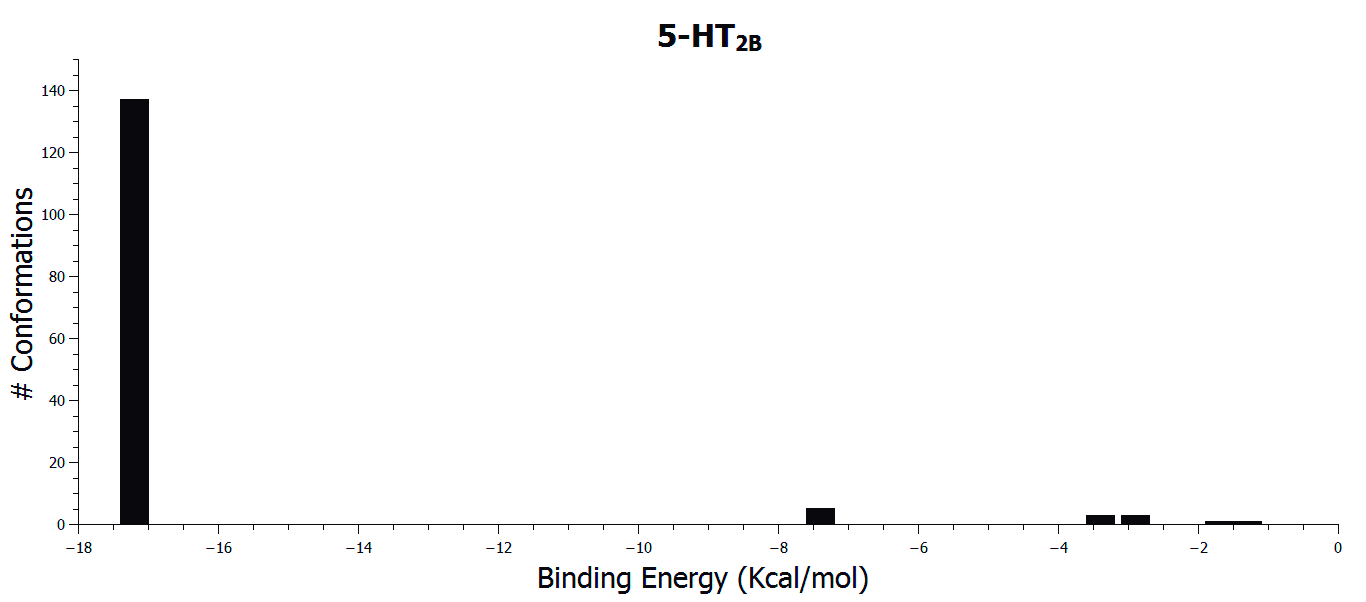

Supplement: S4 Fig — Figure shows the cluster analyses of the AutoDock docking runs of SB-206553 in the drug binding site at the 5-HT2BR. (TIF) [file pone.0134444.s004.tif]

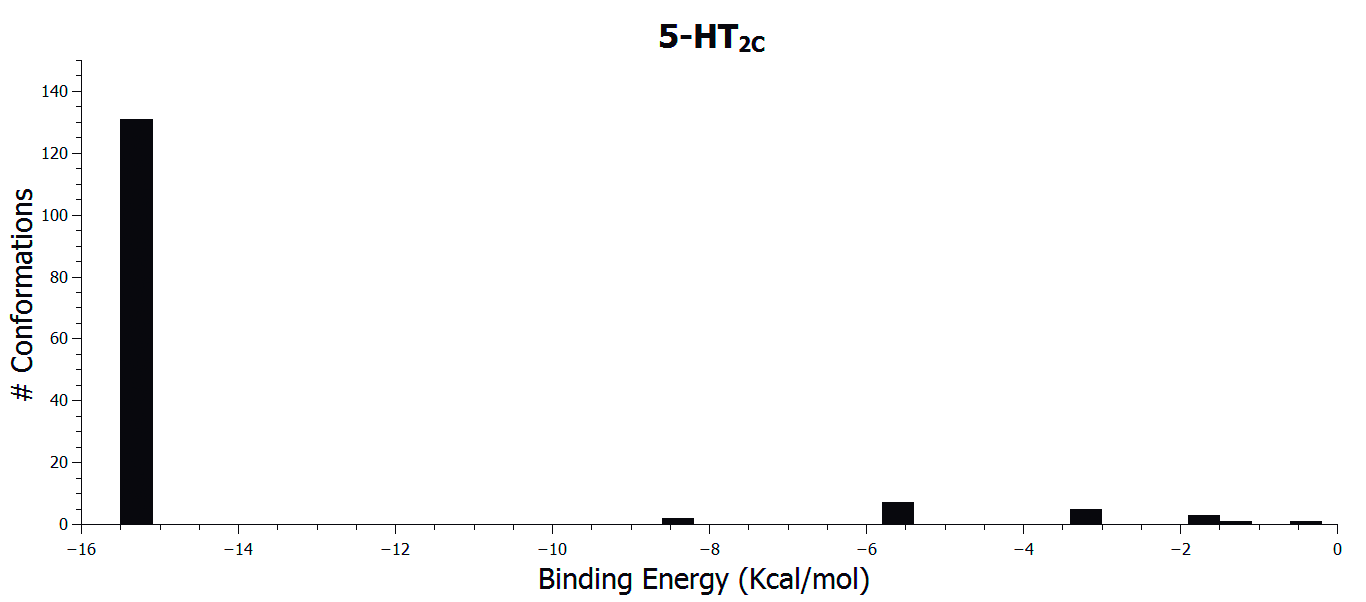

Supplement: S5 Fig — Figure shows the cluster analyses of the AutoDock docking runs of SB-206553 in the drug binding site at the 5-HT2CR. (TIF) [file pone.0134444.s005.tif]

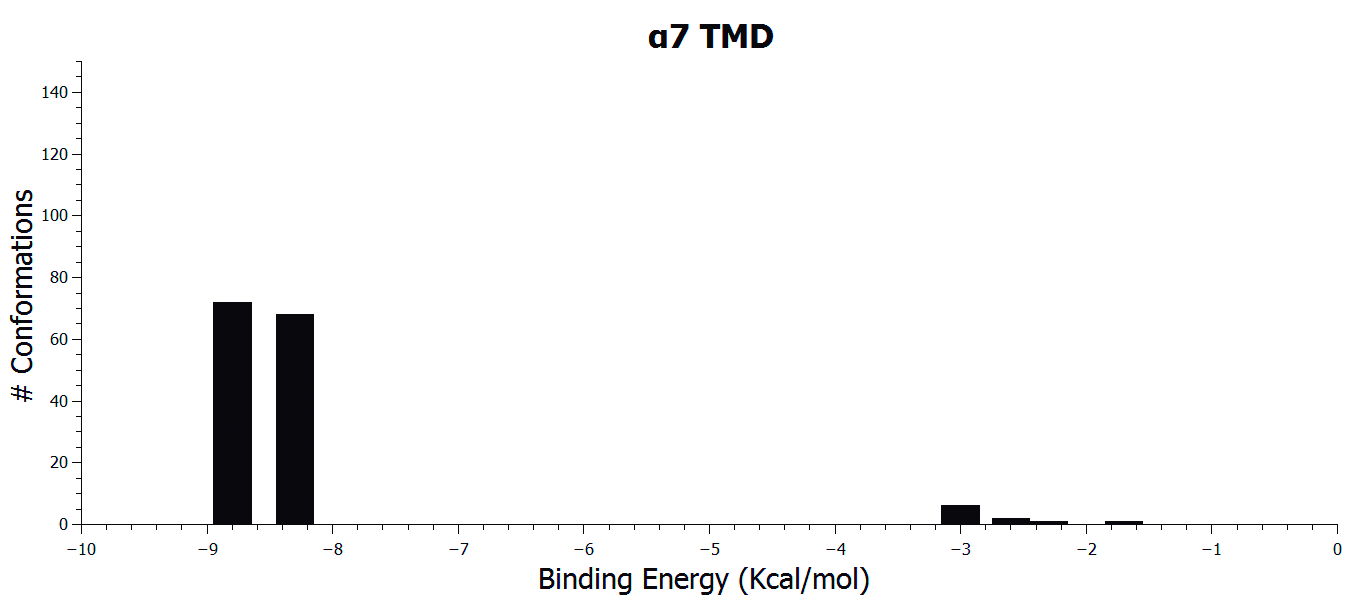

Supplement: S7 Fig — Figure shows the cluster analyses of the AutoDock docking runs of SB-206553 in the drug binding site at the TMD of the α7 nAChR. (TIF) [file pone.0134444.s007.tif]

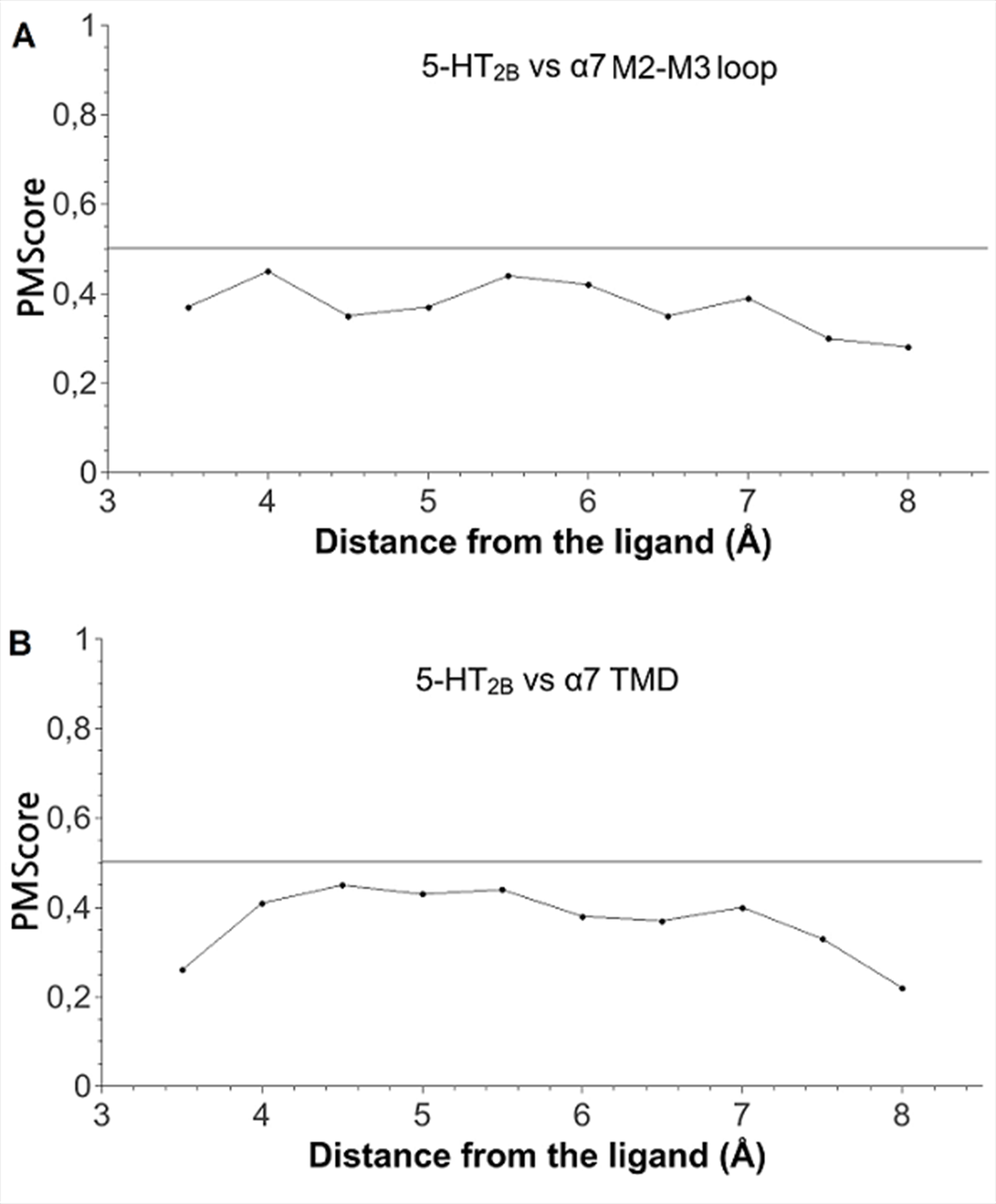

Supplement: S8 Fig — Similarity profiles between the binding sites of SB-206553 docked in the 5-HT2BR and the transmembrane domain (TMD) of the α7 nAChR (A), in the 5-HT2BR and the M2-M3 loop from the α7 nAChR (B), as calculated using PocketMatch. In each case, the horizontal black line indicates PMScore = 0.5. Each point corresponds to the PMScore. (TIF) [file pone.0134444.s008.tif]

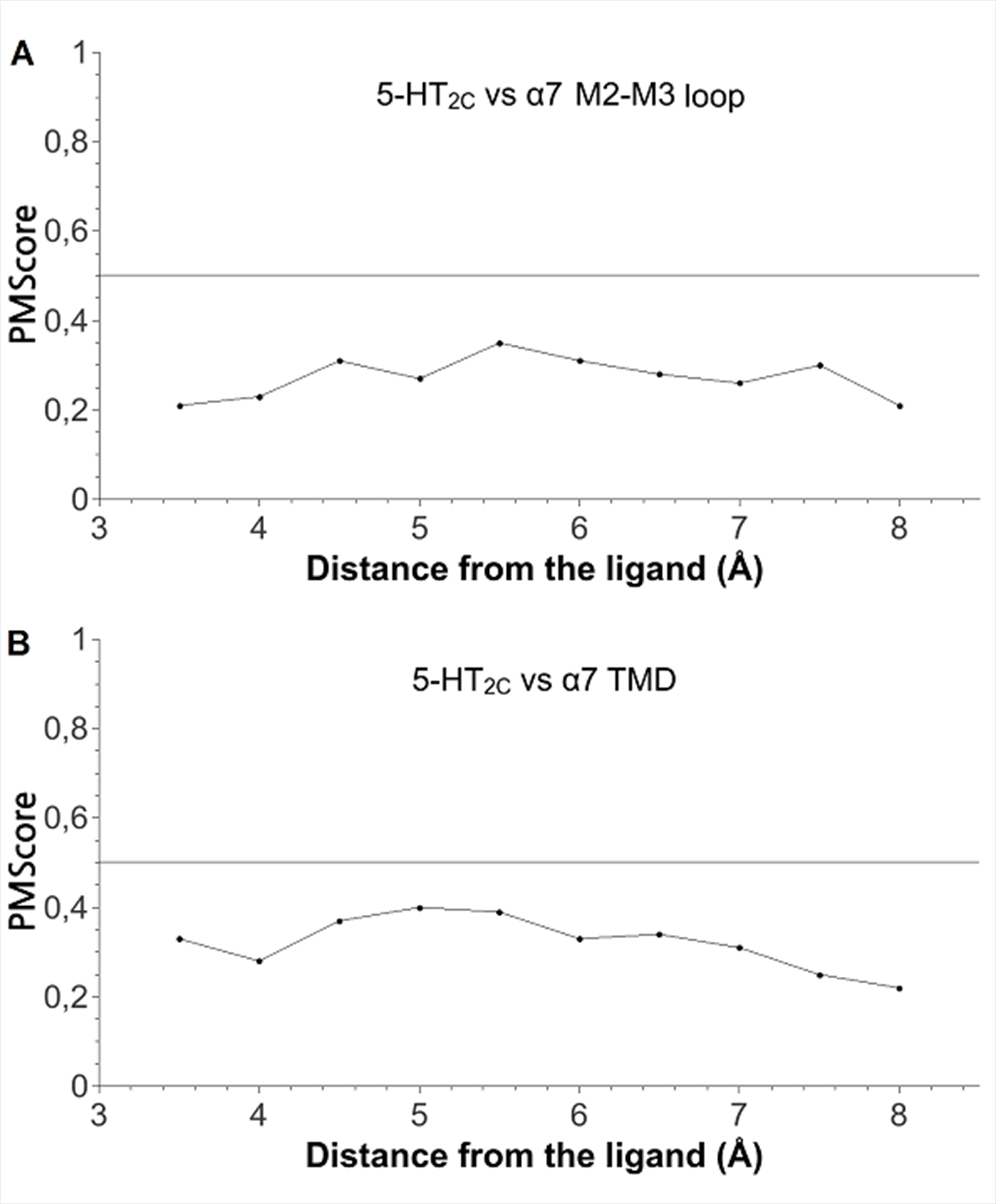

Supplement: S9 Fig — Similarity profiles between the binding sites for SB-206553 docked in the 5-HT2CR and the transmembrane domain (TMD) of the α7 nAChR (A), and in the 5-HT2CR and the M2-M3 loop from the α7 nAChR (B), as calculated using PocketMatch. In each case, the horizontal black line indicates PMScore = 0.5. Each point corresponds to the PMScore. (TIF) [file pone.0134444.s009.tif]
